# Public health round-up

**DOI:** 10.2471/BLT.18.010718

**Published:** 2018-07-01

**Ebola in DRC**

Ring vaccination for Ebola virus disease got underway in the Democratic Republic of the Congo (DRC) at the end of May, targeting those at high risk of infection. Vaccination with rVSV-ZEBOV is one of several interventions in the response, including clinical case management, surveillance and contact tracing and safe burial. This is the first health worker to be vaccinated in the city of Mbandaka on 21 May. bit.ly/2M6aKKi

**Figure:**
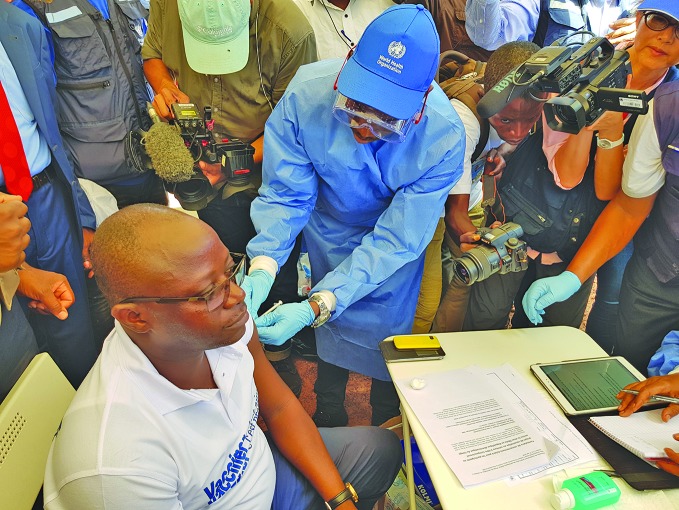
WHO/E. Kabambi

## Therapies for Ebola

An ethics committee in the Democratic Republic of the Congo (DRC) approved the compassionate use of five investigational therapeutics to treat patients with Ebola on 4 June, under an ethical framework that the World Health Organization (WHO) developed in 2016.

Investigational therapeutics are promising chemical compounds or biologic agents that have not been fully tested for clinical safety and efficacy. 

Supplies of ZMapp, Remdesivir, REGN3470-3471-3479, mAb 114 and Favipiravir are already available in the country. 

Clinicians working in Ebola treatment centres will decide which, if any, investigational therapeutic could help their patients. Such treatments can only be used with informed consent from patients and as long as protocols are followed, with close monitoring and reporting of any adverse events.

A group of experts convened by WHO on 16-17 May 2018, agreed that the ethical framework, known as *Monitored emergency use of unregistered and experimental interventions*, could be applied in the current DRC outbreak. 

According to the framework, it may be ethical to offer patients experimental interventions on an emergency basis outside of clinical trials, as long as certain conditions are met in the context of an outbreak with high mortality. 

Conditions are: no proven effective treatment exists, it’s not possible to start clinical studies immediately and data are available providing preliminary support for the intervention’s safety and efficacy at least in laboratory or animal studies. 

ZMapp was tested during the 2014–16 West African outbreak, but the trial did not reach enough participants to produce conclusive evidence of efficacy. 

There are fewer clinical data available for the second therapeutic, Remdesivir (GS-5734), than for ZMapp in patients with Ebola, and no data on Remdesivir’s efficacy in humans beyond anecdotal experience. 

The results from animal studies for REGN3470-3471-3479 are similar to those observed with ZMapp. There are limited human safety data available from a phase 1 study of REGN3470-3471-3479 and these findings suggest that the compound is well tolerated in humans. 

The fourth therapeutic, mAb 114, is at a very early stage of development with data that allowed the start of a phase 1 trial in May 2018. For the fifth therapeutic, Favipiravir, there are limited human data and uncertainties on the required dosing regimen.

http://bit.ly/2sK8LCF

## WHO physical activity plan

A new WHO plan outlines the policies needed to make it easier for people to be physically active and calls on governments to implement these to reduce physical inactivity by 15% by 2030.

The *WHO Global action plan on physical activity and health 2018-2030: more active people for a healthier world,* recommends policies in 20 areas to encourage more activity, including walking, cycling and sport, for example by creating sidewalks and cycle paths.

To support countries to implement the plan, WHO launched a campaign to promote physical activity entitled, *Let’s be active: everyone, everywhere, everyday*.

The campaign follows the 1 June release of six policy recommendations for countries by the Independent High-level Commission on Noncommunicable Diseases.

One in five adults and four of five adolescents (11-17 years) globally do not get enough physical activity. Regular physical activity is key to preventing noncommunicable diseases (NCDs) such as heart disease, stroke and diabetes.

bit.ly/2xU8Ae6

## Health crisis preparedness

WHO and the World Bank Group launched a new initiative on 24 May to strengthen global health security. The initiative combines independent monitoring and reporting on global preparedness for outbreaks and other emergencies.

The Global Preparedness Monitoring Board is co-chaired by Dr Gro Harlem Brundtland, former prime minister of Norway and former WHO Director-General, and Mr Elhadj As Sy, Secretary General of the International Federation of the Red Cross and Red Crescent Societies. Its members will include political leaders, heads of United Nations agencies and leading health experts.

The initiative’s launch comes in the midst of an Ebola outbreak in the Democratic Republic of the Congo, underscoring the need for concerted efforts to tackle such outbreaks and other health crises, and to ensure that all countries have the core capacities required under the International Health Regulations (IHR) (2005).

The Board will monitor preparedness worldwide and issue an annual report on issues that they have identified, as well as noting parallel or duplicative efforts in health crisis preparedness.

The United Nations Secretary-General’s Global Health Crises Task Force, created in response to the 2013-2016 West Africa Ebola outbreak, called for robust monitoring of global health emergency preparedness in July 2017.

Since then, WHO and the World Bank have been working together to establish the new Global Preparedness Monitoring Board. The Board’s secretariat will be hosted by WHO.

bit.ly/2sBaJWR

**Cover photo**

Over 100,000 people are currently displaced from their homes in Ituri province, Democratic Republic of the Congo, as result of violence that erupted in the area. In the town of Bunia, there are two camps housing thousands of internally displaced people. In both camps, Médecins Sans Frontières (MSF) has built toilets and showers, and teams are also helping to ensure the camps have a safe water supply.

**Figure:**
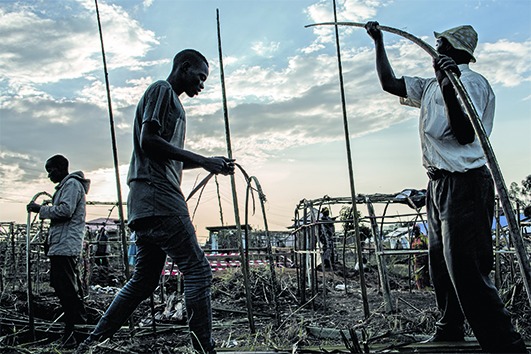
(MSF/ John Wessels)

## Mental health gaps persist

Many countries face shortages of health workers that are trained in providing mental health care and there is a lack of investment in community-based mental health approaches, according to WHO's *Mental health atlas 2017* released last month.

The Atlas is based on data provided by 177 WHO Member States, representing 97% of the world’s population.

Based on these reported data, the number of mental health care workers range from 2 per 100 000 population in low-income countries to more than 70 in high-income countries. An estimated 10% of the global population needs mental health care at any given time.

Respondents from 139 countries reported that mental health policies and plans had been established, but fewer than half of these plans stress a shift from institutional to community-based services. Most do not take a human rights approach allowing people with mental disorders to take part in the decisions concerning them.

Moreover, when mental health plans are made in many countries, these plans are not supported by adequate human and financial resources.

The Atlas measures the extent to which countries are strengthening leadership and governance for mental health; providing comprehensive mental health and social care; implementing strategies to promote mental health and prevent problems, and strengthen evidence and research, as envisaged by WHO’s *Comprehensive mental health action plan 2013-2020*.

bit.ly/2sBlrMS

## Essential diagnostics list

WHO released its first model list of in vitro diagnostics, comprising over 100 laboratory tests that can be used to diagnose a range of health conditions using human specimens, such as blood and urine.

Approximately half of the tests listed are routine basic tests performed at health care facilities.

The remaining tests are for the detection, diagnosis and monitoring of diseases deemed “priority”, such as HIV, tuberculosis, malaria, hepatitis B and C, human papillomavirus and syphilis.

The list will be expanded and updated once a year to include tests needed for noncommunicable diseases, neglected tropical diseases, antimicrobial resistance and outbreaks.

Similar to WHO’s *Model list of essential medicines*, the essential diagnostics list is intended to serve as a reference for countries and a guide for procurement.WHO will provide technical support to countries as they adapt the list to the local context.

bit.ly/2kPiJz0

## New IARC director elected

Dr Elisabete Weiderpass, a Brazilian cancer researcher with Swedish and Finnish citizenship, was elected on 17 May as the new director of the International Agency for Research on Cancer (IARC).

She will take over from incumbent Dr Christopher P. Wild on 1 January 2019 for a five-year term, which can be extended a further five years. Weiderpass was elected by IARC’s Governing Council, which is composed of representatives of IARC’s 26 Participating States and of the WHO Director-General.

Weiderpass holds four posts: head of the Department of Research at the Cancer Registry of Norway, head of the Genetic Epidemiology Group at the Folkhälsan Research Center in Finland, professor of medical epidemiology at the Karolinska Institute in Sweden and professor of cancer epidemiology at the Arctic University of Norway.

She holds adjunct professorship positions in cancer epidemiology in Brazil, China, and the Islamic Republic of Iran, and is a visiting professor in Kuwait.

bit.ly/2Jpf9Gs

## Regional Director appointed

Dr Ahmed Salim Saif Al Mandhari, a public health and medical specialist from Oman, became the new Regional Director for the Eastern Mediterranean on 1 June 2018.

Al Mandhari will serve a term of five years and eight months, completing that of the previous Regional Director for Dr Mahmoud M Fikri, who took office in February of last year and died in October.

bit.ly/2kTMG17

**Looking ahead**

28 July – World Hepatitis Day

26 September – UN General Assembly high-level meeting on ending tuberculosis, New York

9 October – WHO 2018 Symposium on health financing for UHC

30 October – 1 November – WHO Global Conference on Air Pollution and Health, Geneva

